# Editorial: Radiopharmaceuticals, Imaging Techniques and Clinical Applications in Neurodegenerative Diseases

**DOI:** 10.3389/fneur.2019.00962

**Published:** 2019-09-04

**Authors:** Chuantao Zuo, Xiahai Zhuang, Rolf A. Heckemann, Fangyu Peng

**Affiliations:** ^1^PET Center, Huashan Hospital, Fudan University, Shanghai, China; ^2^School of Data Science, Fudan University, Shanghai, China; ^3^Department of Radiation Physics, University of Gothenburg, Gothenburg, Sweden; ^4^Department of Radiology, The University of Texas Southwestern Medical Center, Dallas, TX, United States

**Keywords:** neurodegenerative diseases, molecular imaging, MRI, PET, SPECT

Neurodegenerative diseases are a heterogeneous group of disorders that are characterized by the progressive degeneration of the structure and function of the central nervous system or peripheral nervous system. Common neurodegenerative diseases include Alzheimer's disease (AD) and Parkinson's disease (PD). The prevalence of neurodegenerative disorders is increasing, partly due to the extension of lifespan. Despite intense efforts involving multiple international projects with long-term funding, no treatment that effectively modifies the disease progression is currently available. Critically, no mechanistic model has been developed that comprehensively explains the various changes associated with brain aging and neurodegeneration. Such models are critically needed to develop medical interventions for the prevention, early diagnosis, and treatment of age-related neurodegenerative diseases. Among the most useful approaches to develop an improved understanding of neurodegeneration are structural and functional imaging methods, including magnetic resonance imaging (MRI), positron-emission tomography (PET), and single-photon emission computed tomography (SPECT). With these methods, promising diagnostic biomarkers as well as potential treatment targets for neurodegenerative disease are continuously being identified.

The articles contained in this Research Topic highlight significant advances in molecular imaging of neurodegenerative disease and encourage further investigational efforts in this exciting field. MRI is one of the most widely used imaging techniques in clinical evaluation of neurodegenerative disorders. Owing to its high spatial and contrast resolution, MRI is a useful tool for exploring structural brain changes in neurodegenerative processes associated with aging and disease (1). Zhao et al. adopted structural MRI and diffusion kurtosis imaging (DKI) to characterize gray matter (GM) aging pattern and demonstrate salient macro-microstructure associations during aging. Understanding the associations of GM volume changes and microstructural changes helps to account for the underlying mechanisms of aging and age-related neurodegenerative diseases. Yuan et al. explore the value of multiple visual rating scales based on structural MRI in the diagnosis of AD. Their diagnostic prediction model with a combination of medial temporal lobe atrophy and orbitofrontal cortex shows superior accuracy to any single visual rating scale in the diagnosis of AD.

PET is a useful imaging modality for molecular imaging of metabolic changes in neurodegenerative disease, thanks to its high sensitivity (2). Chen et al. show that brain network and abnormal hemispheric asymmetry analyses reveal differences in glucose metabolic distribution among three dementia subtypes, including AD, PD dementia (PDD), and dementia with Lewy bodies (DLB). Particularly significant are the network properties of the healthy control and AD groups. In addition, differing hub regions were identified in the different dementias. They also identified rightward asymmetry in the hemispheric brain networks of patients with AD and DLB, and leftward asymmetry in the hemispheric brain networks of patients with PDD, which were attributable to aberrant topological properties in the corresponding hemispheres. The study by Zhao et al. provides a quantitative analysis of tau deposits in cognitively normal older adults (CN), mild cognitive impairment (MCI) and AD patients using PET with a novel tracer, ^18^F-AV1451. They quantitatively characterized regional brain tau deposition in participants of different cognitive status, evaluated the correlations between cerebrospinal fluid (CSF) biomarkers or Mini-Mental State Examination (MMSE) and ^18^F-AV1451 PET standardized uptake value ratio (SUVR) as well as evaluated the partial volume effects on ^18^F-AV1451 brain uptake. They found typical deposition of ^18^F-AV1451 tau PET imaging in AD which was strongly associated with cognitive impairment and CSF biomarkers. Though the ^18^F-AV-1451 PET imaging could not differentiate the MCI patients from CN population. In addition, partial volume correction did improve the results of tau deposition and correlation studies in specific brain regions and is suggested to be routinely used in ^18^F-AV1451 tau PET quantification. Jiao et al. found value in combining PET and fMRI in the elderly. While the spatial pattern (significant across-voxel correlation) was similar between ^18^F-FDG PET and resting-state functional MRI (rs-fMRI), their findings show different underlying physiological importance (non-significant across-subject correlation). Their study provides complementary information for identifying underlying mechanisms of brain activity and might enable more comprehensive interpretation of clinical PET-fMRI studies. The findings highlight that substantial benefit can be expected from simultaneous acquisition of the two modalities in hybrid PET-MRI scanners (3).

Various previous studies have shown that PET with a dopamine-analog tracer may aid in the early diagnosis of PD and estimation of disease severity (4). Levodopa-induced dyskinesia (LID) is a highly problematic adverse effect of dopamine replacement drugs used to alleviate akinesia in PD patients. Jeong et al. analyzed serial [I-123] N-ω-fluoropropyl-2β-carbomethoxy-3β-(4-iodophenyl) nortropane (I-123 FP-CIT) SPECT images to investigate changes of dopaminergic innervation during the progression of PD in relation to the development of LID. They found that serial changes of the nigrostriatal dopaminergic innervation in relationship to LID development may predict the development of LID. The findings from this compelling study should be validated on a larger study group.

In summary, articles collected in this Research Topic reflect recent advances in imaging of neurodegenerative diseases, combining multiple imaging modalities and advances in computer science. We hope that this collection will not only stimulate further research studies on structural and molecular imaging of neurodegenerative diseases, but also facilitate discovery of new therapeutic targets for the treatment of Alzheimer's, Parkinson's, and other neurodegenerative diseases.

## Author Contributions

All authors listed have made a substantial, direct and intellectual contribution to the work, and approved it for publication.

### Conflict of Interest Statement

The authors declare that the research was conducted in the absence of any commercial or financial relationships that could be construed as a potential conflict of interest.
